# Abscisic Acid as a Dominant Signal in Tomato During Salt Stress Predisposition to Phytophthora Root and Crown Rot

**DOI:** 10.3389/fpls.2018.00525

**Published:** 2018-04-23

**Authors:** Matthew F. Pye, Sara M. Dye, Renata Sousa Resende, James D. MacDonald, Richard M. Bostock

**Affiliations:** Department of Plant Pathology, University of California, Davis, Davis, CA, United States

**Keywords:** abiotic stress, induced resistance, induced susceptibility, jasmonic acid, *Phytophthora capsici*, salicylic acid

## Abstract

Salt stress predisposes plants to Phytophthora root and crown rot in an abscisic acid (ABA)-dependent manner. We used the tomato–*Phytophthora capsici* interaction to examine zoospore chemoattraction and assessed expression of pathogenesis-related (PR) genes regulated by salicylic acid (SA) and jasmonic acid (JA) following a salt-stress episode. Although salt treatment enhances chemoattraction of tomato roots to zoospores, exudates from salt-stressed roots of ABA-deficient mutants, which do not display the predisposition phenotype, have a similar chemoattraction as exudates from salt-stressed, wild-type roots. This suggests that ABA action during predisposing stress enhances disease through effects on plant responses occurring after initial contact and during ingress by the pathogen. The expression of *NCED1* (ABA synthesis) and *TAS14* (ABA response) in roots generally corresponded to previously reported changes in root ABA levels during salt stress onset and recovery in a pattern that was not altered by infection by *P. capsici*. The PR genes, *P4* and *PI-2*, hallmarks in tomato for SA and JA action, respectively, were induced in non-stressed roots during infection and strongly suppressed in infected roots exposed to salt-stress prior to inoculation. However, there was a similar proportional increase in pathogen colonization observed in salt-stressed plants relative to non-stressed plants in both wild-type and a SA-deficient *nahG* line. Unlike the other tomato cultivars used in this study that showed a strong predisposition phenotype, the processing tomato cv. ‘Castlemart’ and its JA mutants were not predisposed by salt. Salt stress predisposition to crown and root rot caused by *P. capsici* appears to be strongly conditioned by ABA-driven mechanisms in tomato, with the stress compromising SA-and JA-mediated defense-related gene expression during *P. capsici* infection.

## Introduction

Plants rely on an array of phytohormones to coordinate and finely regulate response networks to biotic and abiotic stresses (Grant and Jones, 2009; Bostock et al., 2014). Studies of phytohormone regulation of defense responses in plant–microbe interactions generally have focused on salicylic acid (SA), jasmonic acid (JA), and ethylene (ET). In *Arabidopsis*, SA-mediated defenses are considered deterrents to biotrophic pathogens, whereas JA/ET-mediated defenses deter necrotrophic pathogens (Thomma et al., 2001; Glazebrook, 2005). However, this dichotomy with respect to parasitic strategy may be less clear in other host species (Thaler et al., 2004). While studies of SA and JA/ET signaling have shaped our current understanding of induced resistance mechanisms, consideration of other phytohormones is gaining traction in terms of how multiple stress response pathways overlap as non-linear networks to coordinate plant responses to diverse biotic challengers (Grant and Jones, 2009; Eyles et al., 2010; Bostock et al., 2014). These interactions can be synergistic or antagonistic, or phenotypically neutral if the disease assays cannot discern subtle differences. For example, SA and JA can be antagonistic in certain contexts leading to trade-offs in defense against different attackers (Bostock, 2005; Koornneef and Pieterse, 2008). Similarly, there is substantial evidence that elevated levels of the phytohormone, abscisic acid (ABA), can diminish host resistance (Henfling et al., 1980; Audenaert et al., 2002; de Torres-Zabala et al., 2007; Asselbergh et al., 2008). Nonetheless, ABA enhances resistance in some biotrophic and other interactions (Achuo et al., 2006; Ton et al., 2009b). This parasitic context dependency of ABA action illustrates the challenge in identifying a unifying mechanism to explain ABA’s effects in plant–microbe interactions.

Various root stresses reduce plant water potential and induce ABA accumulation to trigger adaptive biochemical and physiological changes that enable plants to maintain water balance (Taiz and Zeiger, 2010). However, episodic root stresses, even those from which plants fully recover, occur routinely in agricultural and natural systems, transiently elevating cellular ABA concentrations to levels that predispose plants to inoculum densities they would normally resist (Boyer, 1995; Dileo et al., 2010). Disease predisposition from abiotic stress has long been recognized in the plant pathology literature (Yarwood, 1959), and is particularly well-documented in classic studies of root and crown diseases caused by *Phytophthora* spp., where episodes of waterlogging, soil salinity, and drought are important factors in disease development (Duniway, 1977; MacDonald, 1982). Although a role for ABA in conditioning the increased susceptibility during and following stress episodes is recognized, the underlying mechanisms and impacts on host defenses are unresolved (Asselbergh et al., 2008). Furthermore, relatively little attention has been directed at defense-related phytohormone signaling in root–pathogen interactions where the predisposing stresses of water deficit, hypoxia and soil salinity are encountered most directly (Dileo et al., 2010).

Salicylic acid is involved in multiple physiological processes (Vlot et al., 2009), but is perhaps most studied for its role in systemic acquired resistance (SAR) and as a strong inducer of pathogenesis-related (PR) proteins (van Loon et al., 2006). SA biosynthesis in plants occurs by two pathways, one via isochorismate synthase (ICS), and the other via phenylalanine ammonia lyase (Rippert et al., 2009; Vlot et al., 2009). Knockout mutants in the ICS pathway (Catinot et al., 2008) and transgenic plants carrying *nahG* encoding a bacterial salicylate hydroxylase (Gaffney et al., 1993) have reduced SA levels, are highly susceptible to pathogens, have severely reduced levels of PR-proteins, and fail to develop local and systemic resistance (Métraux et al., 1990; Lawton et al., 1995; Seskar et al., 1998; Nawrath and Métraux, 1999; Audenaert et al., 2002). ABA appears to antagonize SA action in defense (Yasuda et al., 2008; Fan et al., 2009).

Jasmonic acid is an oxylipin involved in defense responses against necrotrophic pathogens and insect herbivores, and has been found to have positive or negative interactions with SA depending on the specific host-parasite/pest context (Moons et al., 1997). JA also acts synergistically with the phytohormone ET, and either synergistically or antagonistically with ABA (Robert-Seilaniantz et al., 2007). In soybean leaves, JA levels increase with ABA during dehydration, and a few studies have shown ABA signaling necessary for JA biosynthesis and elaboration of defense responses (Creelman and Mullet, 1995; Adie et al., 2007). In *Arabidopsis*, application of ABA suppresses some JA/ET activated genes such as *PDF1.2*, while JA/ET responsive genes are up-regulated in ABA-deficient mutants such as *aba1* and *aba2* (Anderson et al., 2004). Several JA synthesis mutants are available in tomato, including *defenseless-1* (*def1*), blocked in the conversion of 13-hydroperoxylinoleic acid to 12-oxophytodienoic acid, and *acx1*, a mutant defective in acyl-CoA oxidase (Howe et al., 1996; Li et al., 2005). These mutations result in reduced JA accumulation and pathogen-related transcripts (Schilmiller et al., 2007).

*Phytophthora capsici* is a broad host-range pathogen that can cause significant economic losses in vegetable crops in the Cucurbitaceae, Solanaceae, and Leguminosae families (Lamour et al., 2012). Similar to other soilborne *Phytophthora* species, *P. capsici* causes extensive root and crown rots that are exacerbated by predisposing stresses such as waterlogging and salinity. In a previous study, we imposed acute levels of salt stress on hydroponically grown tomato seedlings prior to inoculation with *P. capsici* to show that predisposition in roots and stems occurred in an ABA-dependent and ET-independent manner (Dileo et al., 2010). In a related study (Pye et al., 2013), we showed that plant activators that engage SA-mediated defenses in tomato induce resistance to the bacterial speck pathogen, *Pseudomonas syringae* pv. *tomato* (*Pst*), both in non-stressed and salt-stressed plants, but not in the case of *P. capsici* where plants exposed to these same treatment regimes displayed similar severity of root and crown rot. The objective of this study was to further assess the impact of salt stress on the infection and colonization of tomato roots by *P. capsici* and to determine if there is discernible interaction between ABA and SA or JA during salinity-induced predisposition. We examined the impact of salt stress on *P. capsici* zoospore attraction and early infection and colonization in tomato roots in wild-type and ABA-deficient mutants. Since ABA can alter the action of SA and JA (Robert-Seilaniantz et al., 2011), we evaluated SA- and JA-deficient tomato plants for altered predisposition phenotypes. In addition, we profiled the expression of hallmark genes for stress adaptation and defense during predisposition onset and recovery and *P. capsici* infection.

## Materials and Methods

### Plant Material and Hydroponic Cultivation

Tomato plants (*Solanum lycopersicum*) of cultivars ‘New Yorker,’ ‘Rheinlands Ruhm,’ or ‘Castlemart’ and mutant or transgenic lines within these backgrounds were used in experiments. ‘New Yorker’ and ‘Rheinlands Ruhm’ are determinate and indeterminate cultivars, respectively, used primarily for fresh market consumption, and ‘Castlemart’ is a determinate, processing cultivar that was bred for the arid growing conditions of California and other regions. In our experimental format, all three cultivars are susceptible to *P. capsici*. ‘New Yorker’ seeds were obtained from a commercial source (Totally Tomatoes, Randolph, WI, United States). The homozygous ABA-deficient mutants *sitiens* and *flacca* were compared with their isogenic, wild-type background, ‘Rheinlands Ruhm,’ and seeds for these were obtained from the C. M. Rick Tomato Genetics Resource Center, University of California, Davis. ‘Rheinlands Ruhm,’ *sitiens*, and *flacca* plants were grown for seed production in the greenhouse. NahG transgenic plants were generated in the ‘New Yorker’ background, similar to the method used by Gaffney et al. (1993). The *nahG* construct containing the transgene salicylate hydroxylase under control of the CaMV 35S promoter in the binary vector pCIB200 was a gift of Syngenta Crop Protection, Inc. SA deficiency of our transgenic line was confirmed previously (Pye et al., 2013). The *acx1* and *def1* mutants in the cv. ‘Castlemart’ background were a gift of Gregg Howe, Michigan State University. Seeds of ‘Castlemart’ were obtained from the C. M. Rick Tomato Genetics Resource Center. Four-week-old plants with two or three true leaves were grown hydroponically as described previously (Dileo et al., 2010; Pye et al., 2013). Experiments were conducted in a growth chamber (150 μmol m^-2^ s^-1^, 16-h photoperiod, 22°C, 70% RH).

### Pathogen Isolates and Culture

A pepper isolate of *P. capsici* (designated “Yolo-1,” from Yolo County, CA, United States; also pathogenic on tomato) was used for most experiments. A *P. capsici* isolate transformed with the green fluorescent protein (GFP) was a gift of Christine Smart and William Fry, Cornell University (Dunn et al., 2013). Wild-type and transformant *P. capsici* strains were maintained on V8 juice agar plates or V8 juice amended with 100 mg/L geneticin (G418; Gibco), respectively. Zoospore inoculum was prepared using methods described previously (Dileo et al., 2010).

### Salinity Stress Treatment and Inoculation

The salt stress regime selected for these experiments was based on prior studies of root stress predisposition (MacDonald, 1982; Bostock et al., 1990; Dileo et al., 2010). The impact of salinity stress differs from other osmotic dehydration stresses primarily in that salt-stressed plants are additionally exposed to abnormally high extracellular concentration of ions such as sodium and chloride (Bartels and Sunkar, 2005). The inclusion of calcium helps to mitigate the confounding toxicity caused by sodium and emphasizes the osmotic facet of salinity stress, which is likely applicable to other dehydration stresses (Cramer, 2002). Plants were subjected to salt stress by replacing the 0.5X Hoagland’s solution with 0.2 M NaCl and 0.02 M CaCl_2_ for 18 h. Plants were returned to 0.5X Hoagland’s solution, allowed to regain turgor and recover for 2 h, and then inoculated with zoospores of *P. capsici* (10^4^ or 10^5^ ml^-1^, as indicated).

### Zoospore Attraction

To determine whether there was an effect on zoospore motility and chemotaxis, a microcapillary swim-in assay similar to that described by Morris and Ward (1992) was used with exudates collected from tomato roots. Following 18 h salt stress, tomato roots of uniform volume were rinsed in deionized H_2_O and transferred to tubes containing 2 ml of deionized water. Exudates were allowed to accumulate for 2 h, tomatoes were then removed, and the exudates were vortexed and immediately loaded into 1 μl microcapillary tubes (Drummond Scientific, Broomall, PA, United States). Exudate-loaded microcapillaries were placed into 15 cm petri dishes with one end submerged in a 500 μl droplet of 5 × 10^5^ zoospores ml^-1^. Microcapillaries were photographed under a dissecting microscope 15 min after being placed into the zoospore suspension. Zoospore attraction was determined as the proportion of the microcapillary’s inside diameter blocked by encysted zoospores and scored on a 0–5 rating scale (zoospore attraction rating scale, ZARS; **Figure 2**).

### Confocal Microscopy

The *P. capsici*-GFP transformant was visualized 24 and 48 hours post inoculation (hpi) in tomato roots using a Leica TCS SPE confocal system (Leica Microsystems GmBH, Germany). Following infection and just prior to microscopy, roots were dipped into a 10 μg/ml solution of propidium iodide (PI, Sigma) for 30 s and rinsed in sterile water before mounting on microscope slides (Huang et al., 1986). GFP was excited at 488 nm and emission was collected between 510 and 550 nm. PI was excited at 534 nm and emission was collected between 600 and 650 nm. Laser power was set to 50% with a gain of 800–900 for both the 488 nm and 534 nm channels. Final images were composites of five Z steps through root tissues approximately 40 μm in depth.

### Pathogen DNA Quantitation and Gene Expression Profiling in Infected Host Tissue

To estimate the progression of *P. capsici* colonization in tomato seedlings by qPCR, *nahG, def1*, and *acx1* plants and wild-type plants of their corresponding backgrounds (cvs. ‘New Yorker’ and ‘Castlemart’) were frozen in liquid N_2_ at 48 hpi, and stored at -80°C until extraction and analysis. Samples for quantitation of *P. capsici* DNA were extracted and analyzed using the method described in Dileo et al. (2010). For gene expression analyses, RNA was extracted from tomato seedlings using RNeasy Plant Mini kits according to the manufacturer’s instructions (Qiagen, Valencia, CA, United States). Samples were obtained from roots pooled from five plants, with three samples for each treatment in each experiment. Extracts were treated with Dnase I (Fermentas) to remove genomic DNA contaminants. Intact 25s and 18s ribosomal RNA bands were visualized by gel electrophoresis (D’Ambrosio et al., 2004). cDNA stock solutions were prepared with the iScript cDNA synthesis kit (Bio-Rad, Hercules, CA, United States). A complete list of target genes and primers can be found in **Table 1**. Gene expression was quantified with a 7500 FAST Real-time PCR thermocycler (Applied Biosystems, Foster City, CA, United States), using SsoFAST EvaGreen Supermix with low Rox (Bio-Rad, Hercules, CA, United States). Relative quantities were determined using the ΔΔCT method, normalizing against cyclophilin (*Cyp*, M55019.1) and uridylate kinase (*UK*, SGN-U566261).

**Table 1:** Real-time qPCR primers used in this study.

Name	Sequence	Product length
*Cyp*	5′GGCCAATTCTGGACCTAACA′3	134 bp
	5′CATGTTCCATAGAGCGGACA′3	
*UK*	5′GCTGTTTTTGCCCCATCTAA′3	154 bp
	5′CATCGTTTTGCTGCTGAAGA′3	
*Phytophthora capsici* target (for quantifying colonization)	5′TTTAGTTGGGGGTCTTGTACC3′	452 bp
	5′CCTCCACAACCAGCAACA3′	
*TAS14*	5′AGATGGCACAATACGGCAAT′3	174 bp
	5′ACCAGTACCCATGCCTTGAG′3	
*NCED1*	5′CTGCTTCTTCCCAAGCATTC′3	176 bp
	5′ACCTGTTCCACCACAAGGAC′3	
*P4*	5′AGGTGACACTATAGAATAAACAATGGGTGGTGGTTCAT′3	143 bp
	5′GTACGACTCACTATAGGGATAGCAACATGTCAGAAATAGACGA′3	
*PI-2*	5′CCCACGTTCAGAAGGAAGTC′3	142 bp
	5′TGAACGGGGACATCTTGAAT′3	
*13-LOX (TomLOXD)*	5′TTGTGCCTGAAAAAGCAGTG′3	141 bp
	5′GTTCTAGCGCGACATTCCTC′3	
*13-AOS (LeAOS1)*	5′GGGGCTAAACTCCACAGTCA′3	147 bp
	5′TGCTACCGGAGGTTCAATTC′3	


*For each pair, the forward primer is listed first followed by the reverse primer. See text for further description.*

### JA-Treatment Experiments

Jasmonic acid was generated by base hydrolysis of methyl jasmonate [3-oxo-2-(2-pentenyl)cyclopentaneacetic acid, methyl ester, 95% purity; Sigma-Aldrich] according to the procedure of Farmer et al. (1992). The experimental treatment sequence was as follows. Roots of hydroponically grown tomato seedlings (cv. ‘New Yorker’) were immersed for 72 h in a solution of JA (25 μM in 0.5X Hoaglands, final concentration of immersive solution). Seedlings were removed from the JA solution and transferred to fresh 0.5X Hoaglands for 48 h, and then exposed to salt stress for 18 h as described above. After a 2 h recovery in 0.5X Hoaglands, the roots were inoculated with 1 × 10^4^ zoospores/ml of *P. capsici*. Roots were then collected at 24 hpi for gene expression analyses as described above, with samples obtained from roots pooled from five plants and three samples analyzed for each treatment. JA at 25 μM was selected because higher concentrations (50–100 μM) were slightly phytotoxic in our experimental format. Appropriate controls (i.e., no JA, no salt, no inoculation, and various combinations thereof, as indicated) were included.

### Statistical Analyses

Disease assays in ‘New Yorker,’ ‘Rheinlands Ruhm,’ and ‘Castlemart’ backgrounds were performed three times, with five replicates for each treatment within each experiment. For ordinal data and for qPCR data that typically did not satisfy the analysis of variance (ANOVA) criterion for normality, the Wilcoxon rank sums or Kruskal–Wallis tests were used for means comparisons. Gene expression time courses were performed twice. When data satisfied the criterion for normality, ANOVA and the Dunnett’s test or Student’s *T*-test were used for means comparisons. Analyses were performed with JMP Pro software (SAS, Inc.).

## Results

### *P. capsici* Zoospore Attraction and Infection in Predisposed Tomato Roots

**FIGURE 1:**
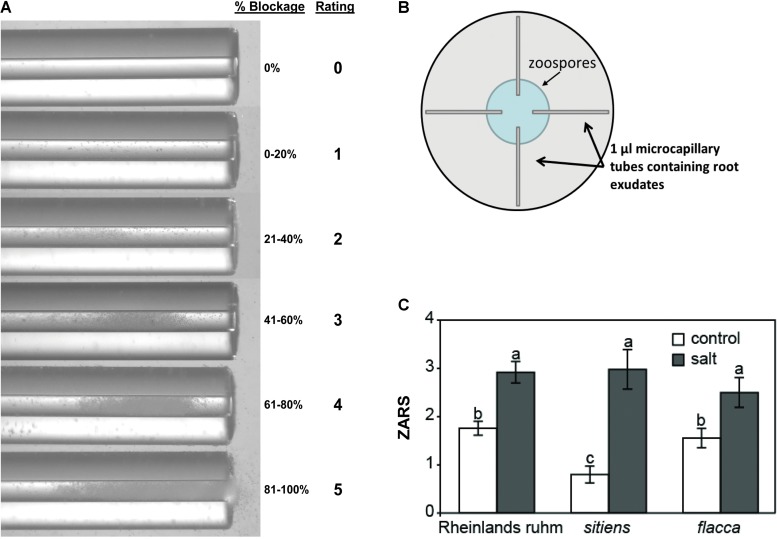
Attraction of *P. capsici* zoospores to the root exudates of tomatoes. Root exudates were collected from non-stressed (control) and salt-stressed (0.2 M NaCl/0.02 CaCl_2_) roots of wild-type ‘Rheinlands Ruhm’ (RR) and the ABA-deficient mutants, *sitiens* and *flacca.* **(A)** Microcapillary tubes with root exudates showing varying degrees of blockage of the tube by zoospores. The zoospore attraction rating scale (ZARS) is indicated on the right. **(B)** One microliter microcapillary tubes were filled with exudates and then one end submerged in a droplet of *P. capsici* zoospores (5 × 10^5^ ml^-1^) in a 15 cm diameter petri dish as shown. Results were scored 15 min later. **(C)** ZARS values for each treatment and tomato genotype. Values are the means ± SE from three experiments, with five samples each from a separate seedling for each treatment within an experiment (*n* = 15). Letters indicate significant differences among treatment means at *P* = 0.05 by the Kruskal–Wallis test as performed in JMP Pro 13.0. There is also a significant difference in attraction between the salt and the control treatments of ‘Rheinlands Ruhm’ and *sitiens*.

A brief episode of salt stress applied prior to inoculation of tomato seedlings with zoospores of *P. capsici* results in infections of greater severity and a classic predisposition phenotype (**Supplementary Figure S1**). Previously, increased zoospore attraction was observed in salt-stressed chrysanthemum roots relative to non-stressed roots (MacDonald, 1982). To determine if salt-stress enhances the attraction of tomato roots to zoospores and whether ABA influences this, we used a quantitative chemotaxis choice assay to compare exudates from non-stressed and salt-stressed tomato roots. Exudates collected from ABA-deficient *flacca* and *sitiens* mutants and their background wild-type ‘Rheinlands Ruhm’ roots following salt stress were significantly more attractive to *P. capsici* zoospores than exudates collected from non-stressed roots. However, exudates from the ABA-deficient mutants, *sitiens* and *flacca*, were equally attractive as those collected from ‘Rheinlands Ruhm’ (**Figure 1**). ABA alone was not a chemoattractant in this assay, having a ZARS value of 0, the same as deionized water.

**FIGURE 2:**
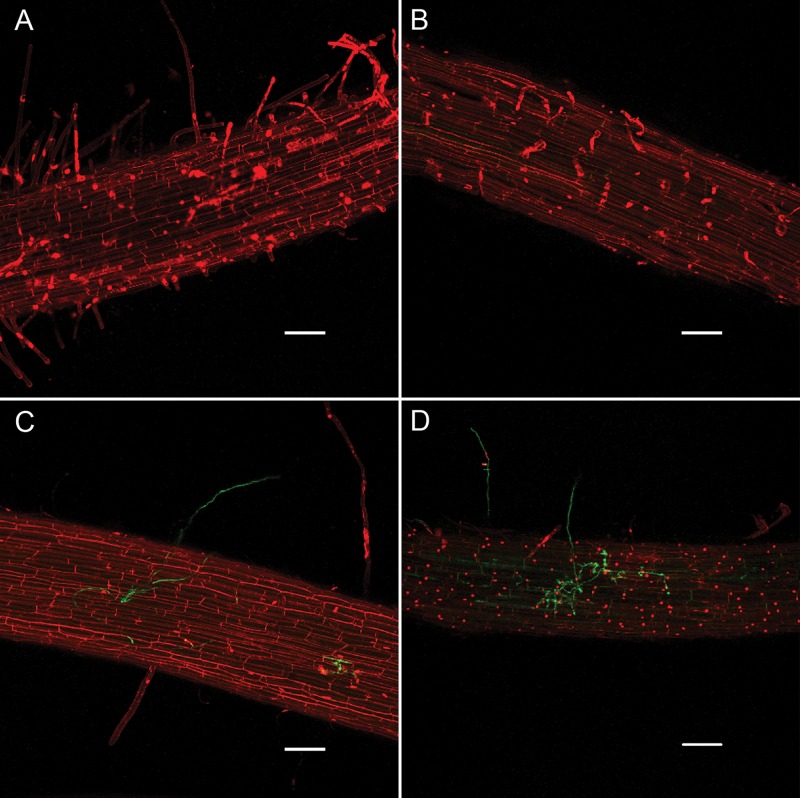
Infection of ‘New Yorker’ tomato roots by *P. capsici*-GFP (green fluorescence) visualized by confocal microscopy with propidium iodide staining (red fluorescence), 24 hpi. **(A)** Non-stressed non-inoculated tomato roots. **(B)** Salt-stressed (0.2 M NaCl/0.02 CaCl_2_), non-inoculated tomato roots. **(C)** Non-stressed, inoculated tomato roots (*P. capsici* at 10^4^ zoospores ml^-1^). **(D)** Salt-stressed (0.2 M NaCl/0.02 CaCl_2_) inoculated tomato roots (*P. capsici* at 10^4^ zoospores ml^-1^). Bars indicate 100 μm.

We used confocal microscopy to further characterize root infections under our experimental regime to determine if salt stress of the host prior to inoculation causes *P. capsici* to change its infection and colonization strategy. Examination of roots inoculated with a *P. capsici-*GFP strain 24 hpi revealed haustoria in host cells deep within the root tissue (**Supplementary Figure S2**). Haustoria were observed in both salt-stressed and non-stressed roots, with the only apparent microscopic distinction between the treatments during the course of observation being the greater extent of colonization in salt-stressed roots. Propidium iodide (PI), which stains nuclei in dead or dying cells, was used as a vital stain to assess root cell viability under the various treatments. Non-inoculated roots in the non-stressed and salt-stressed treatments were similar in appearance, with occasional PI-staining of nuclei (**Figures 2A,B**). There was non-specific staining of plant cell walls by PI in all treatments, which is common due to the exclusion of the dye from membranes of living cells that makes outlines of the cells visible. Inoculated, non-stressed roots were mostly intact with limited instances of PI staining of nuclei (**Figure 2C**), while inoculated, salt-stressed roots contained numerous PI-stained nuclei (**Figure 2D**). In both treatments, root tips and the bases of lateral roots were the most colonized regions.

### ABA-Related Gene Expression During Predisposing Salt Stress and *P. capsici* Infection

**FIGURE 3:**
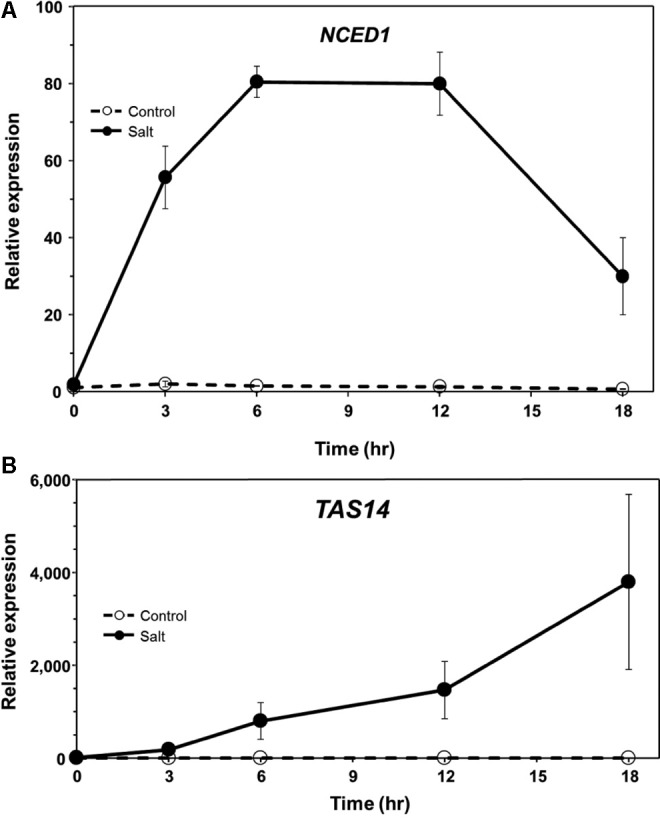
ABA-related gene expression in ‘New Yorker’ tomato seedling roots during the onset of salt stress. **(A)** Time course of *NCED1* and **(B)** *TAS14* expression in non-stressed (control) and salt-stressed tomato roots (0.2 M NaCl/0.02 CaCl_2_). *NCED1* and *TAS14* expression was normalized against *Cyp* and *UK*. Values are the means ± SE from two experiments, with roots from five plants pooled per sample and three samples analyzed for each treatment mean per time point.

**FIGURE 4:**
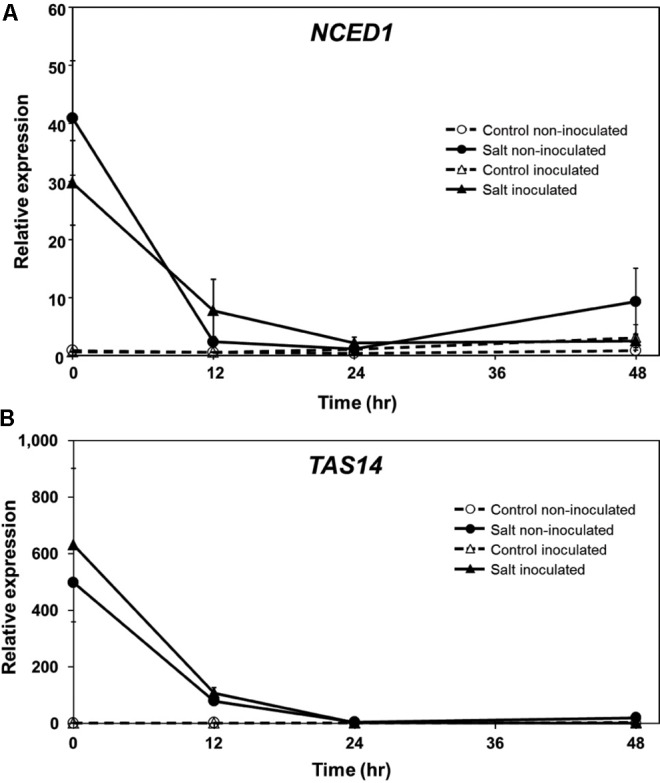
ABA-related gene expression in inoculated ‘New Yorker’ tomato seedling roots following an episode of salt stress. **(A)** *NCED1* and **(B)** *TAS14* expression in non-stressed (control) roots and in roots after 18 h exposure to 0.2 M NaCl/0.02 CaCl_2_, with (triangles) and without (circles) inoculum (*P. capsici* at 2 ml of 10^4^ zoospores ml^-1^). *NCED1* and *TAS14* expression was normalized against *Cyp* and *UK*. Values are the means ± SE from two experiments, with roots from five plants pooled per sample and six samples analyzed for each treatment mean per time point. Note that seedlings were removed from the salt stress and returned to 0.5X Hoaglands during the course of analysis.

In a previous study, we found that ABA levels in tomato roots increase rapidly following exposure to salt stress and during the onset of predisposition, and then decline to near pre-stress levels (Dileo et al., 2010). To determine if the expression of genes associated with ABA synthesis and response follows a similar course during stress onset and recovery, *NCED* and *TAS14* were monitored by qPCR in tomato roots. *NCED* encodes the 9-*cis*-epoxycarotenoid-dioxygenase (EC 1.13.11.51), a critical step in ABA biosynthesis and generally considered to be rate-limiting (Qin and Zeevaart, 1999; Thompson et al., 2007). *TAS14* (X51904.1) is a tomato dehydrin gene that is induced by salt stress and ABA, but not by cold or wounding, and serves as a salt stress-induced marker of ABA responses in tomato (Godoy et al., 1990). *NCED1* expression increased rapidly in tomato roots following salt exposure in a manner that generally corresponded with ABA measurements reported previously (see Figure 5 in Dileo et al., 2010), and returned to pre-stress levels similar to ABA (**Figure 3A**). Salt challenge of tomato roots induced *TAS14* within 3 h after immersion of the roots in the salt solution, with maximum expression as much as ∼4,000-fold above the initial basal expression (**Figure 3B**). *NCED1* gene expression levels returned to basal levels 24 h following removal of the roots from the salt treatment (**Figure 4A**), whereas *TAS14* gene expression levels from the same plants returned to pre-stress values within 12 h of salt removal (**Figure 4B**). The changes in TAS14 expression were limited to salt-stressed roots, as baseline expression in non-stressed roots was at or below the sensitivity of our analytical platform. *P. capsici* infection in either salt-stressed or non-stressed plants did not appear to influence *NCED1* and *TAS14* expression.

### Defense-Related Gene Expression Following Salt Stress and Disease Onset

**FIGURE 5:**
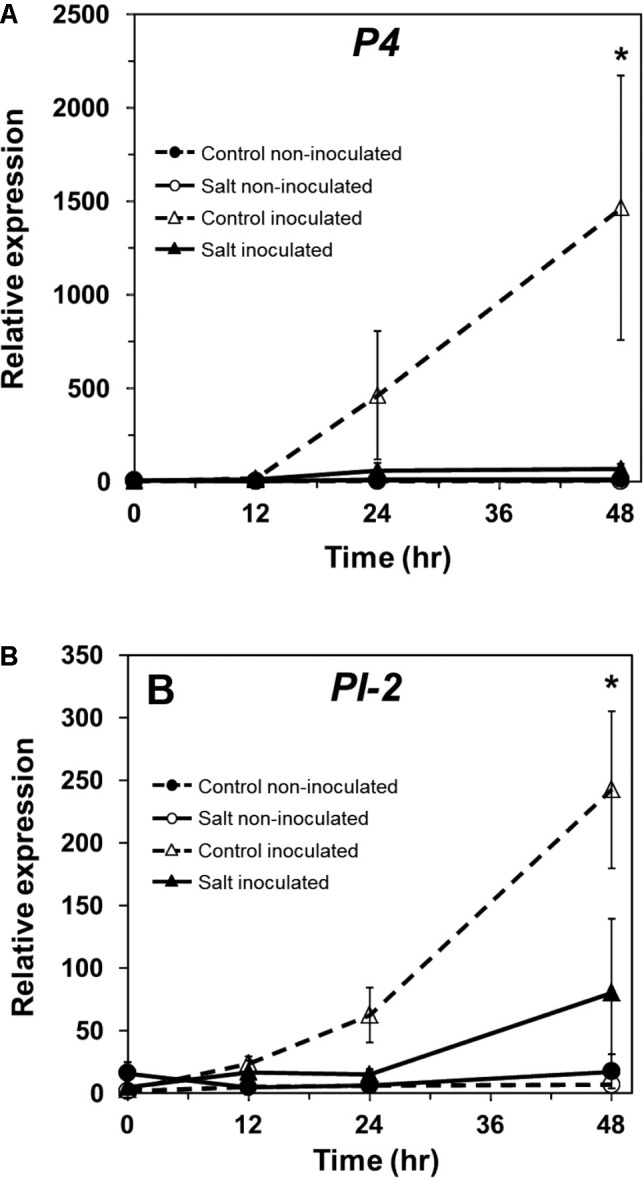
Pathogenesis-related protein gene expression in inoculated and salt-stressed ‘New Yorker’ tomato seedling roots. **(A)** Time course of *P4*, and **(B)**, *PI-2* expression in non-stressed (control) and 18 h salt stressed (0.2 M NaCl/0.02 CaCl_2_) roots not inoculated or inoculated with *P. capsici* at 10^4^ zoospores ml^-1^. *P4* and *PI-2* expression was normalized against *Cyp* and *UK*. Values are the means ± SE from two experiments, with roots from five plants pooled per sample and six samples analyzed for each treatment mean per time point. Asterisks indicate significant differences among treatment means by the Wilcoxon rank sums test. For *P4*, χ^2^ = 14.06, *P* = 0.003; for *PI-2*, χ^2^ = 12.19, *P* = 0.007.

In tomato, *P4* (M69247.1), a *PR-1* ortholog, serves as a marker for induction of the SA pathway (Fidantsef et al., 1999; Uehara et al., 2010). *P4* transcript accumulation was measured in non-stressed and salt-stressed ‘New Yorker’ tomato roots following inoculation with *P. capsici. P4* was induced only in plants inoculated with *P. capsici* (**Figure 5A**). Plants that had been salt-stressed prior to inoculation had significantly lower levels of *P4* transcripts relative to non-stressed, inoculated plants (**Figure 5A**). *P4* expression remained suppressed even at 48 hpi in salt-stressed, inoculated plants.

In tomato, proteinase inhibitor II (*PI-2*, K03291.1) is a wound- and pathogen-inducible marker of JA responses (Farmer and Ryan, 1992; Hondo et al., 2007). *PI-2* showed a similar pattern of expression as *P4* in our experimental regime and was induced only in *P. capsici*-inoculated plants (**Figure 5B**). Prior salt stress resulted in significantly reduced *PI-2* gene expression throughout the period of observation (48 hpi; **Figure 5B**). Salt stress alone did not induce *P4* or *PI-2* expression.

### Assessment of Predisposition to Phytophthora Root and Crown Rot in SA and JA-Modified Tomato Plants

**FIGURE 6:**
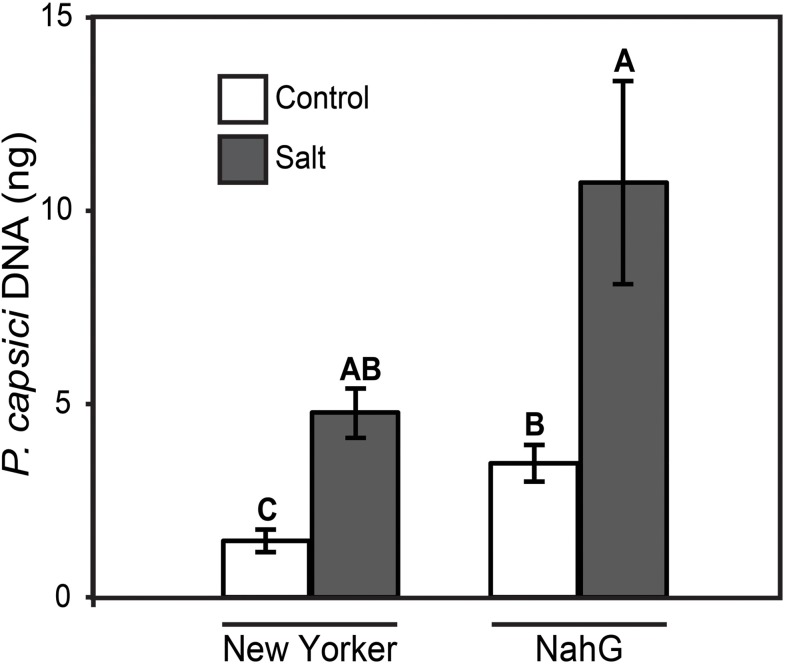
*P. capsici* colonization 48 hpi on ‘New Yorker’ (WT) and NahG (in “New Yorker’ background) tomato seedlings non-stressed (control) or salt stressed with 0.2 M NaCl/0.02 M CaCl_2_ for 18 h. Colonization estimated by qPCR of pathogen DNA. Letters indicate significant differences at *P* = 0.05 (*T*-test). Values are the means ± SE from three experiments, with five samples, each from a separate seedling, for each treatment within each experiment (*n* = 15).

To determine if SA and JA influence the severity of disease susceptibility induced by salt-stress, tomato plants altered in SA levels (*nahG* transgenic) and JA synthesis (*acx1* and *def1* mutants) were evaluated in the predisposition assay. NahG and WT (cv. ‘New Yorker’) tomatoes both displayed enhanced susceptibility following salt stress, but NahG plants had significantly higher basal susceptibility to *P. capsici* even without salt stress (**Figure 6**). Nonetheless, the proportional increase in *P. capsici* colonization in salt-treated plants relative to non-salted plants was similar in both the WT (3.2-fold increase) and NahG (3.1-fold increase) tomato genotypes.

‘Castlemart’ tomatoes, and the *acx1* and *def1* mutants within this genetic background, unlike other tomato genotypes we have used in predisposition studies, did not display a predisposition phenotype under our treatment regime (**Supplementary Figure S3**). Colonization of these plants by *P. capsici* trended less in the salt-treated seedlings, and significantly less (*P* = 0.032) in salt-treated *acx1* seedlings compared to non-salted plants (**Supplementary Figure S3B**). This was unexpected, rendering results with the *def1* and *acx1* mutants inconclusive relative to the issue of JA action in predisposition.

**FIGURE 7:**
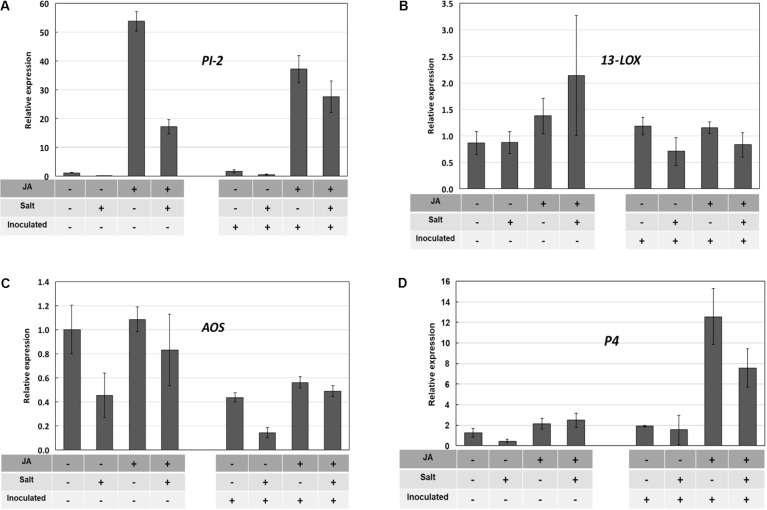
Pathogenesis-related (PR) and JA-synthesis gene expression in ‘New Yorker’ tomato roots in response to JA, salt stress, and inoculation with *P. capsici*. Treatment combinations are indicated by “+” and “–” below each bar. Bars indicate relative gene expression determined by RT-qPCR (means and SE from three determinations are indicated; selected means were compared by Wilcoxon rank sums test). The treatment sequence was: JA or water (72 h) recovery (48 h) salt or no salt (18 h) recovery (2 h) inoculated or non-inoculated (24 h) collect roots for RT-qPCR. See Section “Materials and Methods” for additional detail. **(A)** *PI-2* expression; JA strongly induced *PI-2* expression (χ^2^ = 15.6, *P* < 0.0001), salt suppressed the JA induction (χ^2^ = 3.15, *P* = 0.076) **(B)** *13-LOX* expression; **(C)** *13-AOS* expression; salt reduces AOS expression (χ^2^ = 4.03, *P* = 0.045) **(D)** *P4* expression; JA potentiates *P4* expression in inoculated roots (χ^2^ = 3.00, *P* = 0.083).

Without suitable JA-deficient mutants available to this study, we then sought to determine whether exogenous JA could alter or override the salt stress inhibition of *PI-2* gene expression using ‘New Yorker’ seedlings, which display a consistent and clear predisposition phenotype. Treatment of roots with exogenous JA (25 μM) strongly induced *PI-2* transcripts, with salt treatment reducing transcript accumulation (**Figure 7A**). The *PI-2* expression pattern was similar in the inoculated seedlings pretreated with JA and/or salt. The tomato *13-LOX* and *13-AOS* genes encode key enzymes in JA biosynthesis (Mosblech et al., 2009). *13-LOX* expression at the time of sampling was not significantly affected by any treatment (**Figure 7B**). Although *AOS* transcript levels were relatively low in all treatment combinations, salt stress reduced *AOS* expression by more than half in both non-inoculated and inoculated seedling roots (**Figure 7C**). This reduction was partially offset by JA pre-treatment. *P4* expression was not induced by JA, salt or their combination; however, inoculation with *P. capsici* following JA treatment resulted in a strong induction of *P4* transcripts (**Figure 7D**).

## Discussion

Previous research in our laboratory demonstrated that tomato seedling roots and crowns became highly susceptible to *P. capsici* following a brief exposure of the roots to salt stress (Dileo et al., 2010) (**Supplementary Figure S1**). These plants generally regained turgor during the course of the stress treatment, but remained in a predisposed state in the absence of visible stress symptoms for up to 24 h following removal from the salt. The salt stress effect on disease appears to operate through an ABA-dependent mechanism, as evidenced by the loss of predisposition in ABA-deficient mutants and partial complementation with exogenous ABA to restore the predisposition phenotype (Dileo et al., 2010). Salinity stress also has been shown to make roots more attractive to *Phytophthora* zoospores (MacDonald, 1982). In the present study, chemoattraction of *P. capsici* zoospores to exudates from salt-stressed roots was significantly greater than to exudates from non-stressed roots. However, exudates from salt-stressed roots of wild-type tomato plants and ABA-deficient mutants were equally attractive (**Figure 1**). Thus, differences in root attraction to zoospores cannot explain the differences in disease severity between wild-type and ABA-deficient plants. These results reinforce our view that the determinative effects of stress-induced ABA in predisposition occur during infection, invasion and colonization, rather than during pre-infection events related to root exudation, zoospore attraction and initial contact with the root (Swiecki and MacDonald, 1988). Our results also affirm an earlier study on salinity-induced susceptibility to Phytophthora root rot that pointed to a strong effect of the stress on host defenses (MacDonald, 1984).

*P. capsici* is a hemibiotroph, establishing haustoria in host cells during the early stages of infection, and then necrotizing host tissue as the infection progresses (Lamour et al., 2012). Confocal imaging revealed the presence of haustoria in infected tomato roots that appeared as simple protrusions into root cells (**Supplementary Figure S2**), closely resembling those described in the literature for *Phytophthora* haustoria (Hwang et al., 1989; Lee et al., 2000). After reviewing dozens of *P. capsici* infections in non-stressed and salt-stressed roots, we concluded that haustoria are present in both treatments. Therefore, it does not appear that *P. capsici* alters its fundamental infection strategy in salt-stressed tomato roots. The only clear distinction apparent between treatments was the increased rate of colonization, as reflected in greater abundance of hyphae in the salt-stressed roots relative to the controls. While the pathogen’s infection strategy does not appear to change, based on microscopic examination, it is possible that *P. capsici* alters its strategy in other ways, such as the timing or pattern of display of effectors. We attempted to measure expression of putative and known *P. capsici* effector genes believed to correspond to the switch from biotrophy to necrotrophy (Kelley et al., 2010). Pathogen RNA proved difficult to recover during early infection and later as plant tissues died, and so we were unable to detect alterations in effector expression as a function of treatment. Transcriptome analyses using deep sequencing as reported in a study of *P. capsici* on tomato leaves may prove to be better able to address this question (Jupe et al., 2013).

Endogenous ABA levels are tightly regulated in the plant by balancing biosynthesis, catabolism and conjugation (Tian et al., 2004; Nambara and Marion-Poll, 2005; Szepesi et al., 2009). *NCED1* expression in roots during the 18 h salt stress treatment (**Figure 3A**) generally corresponded with salt-induced ABA accumulation that we reported in our previous study (Dileo et al., 2010). Similar findings in *Phaseolus vulgaris* showed stress-induced expression of *NCED*, with accumulation of NCED protein and ABA occurring within a 2 h window (Qin and Zeevaart, 1999). While stimuli have been described that up-regulate *NCED1* gene expression, relatively little information is available regarding mechanisms for its down regulation. In drought-stressed *Arabidopsis*, ABA production and expression of *NCED3* (homologous to tomato *NCED1*) is correlated with the level of available carotenoid substrates (Tian et al., 2004). *NCED1* expression in tomato roots may diminish as ABA levels decline or as external stresses are removed. Possible post-transcriptional and/or post-translational regulation of *NCED1/*NCED cannot be ruled out, as suggested for regulation of *AAO* (abscisic aldehyde oxidase), the terminal step in ABA synthesis (Xiong et al., 2001; Seo and Koshiba, 2002). Following an episode of salt stress and inoculation with *P. capsici*, *NCED1* transcript levels returned to pre-stress levels in tomato roots and remained at basal levels in all treatments throughout the 48 h infection time course (**Figure 4A**). However, we saw no evidence for *NCED1* induction or ABA accumulation during infection with *P. capsici*. This is in contrast to *Arabidopsis* infected by *Pst*, which induces *AtNCED3* and ABA accumulation in leaves (de Torres-Zabala et al., 2007).

Expression of *TAS14*, which encodes a tomato dehydrin, is triggered by osmotic stress and ABA (Godoy et al., 1990). When overexpressed in tomato, *TAS14* confers partial drought and salinity tolerance (Muñoz-Mayor et al., 2012). In our study, *TAS14* increased rapidly after salt stress onset and remained elevated throughout the course of the stress treatment. Similar to *NCED1*, *TAS14* did not show altered expression following *P. capsici* infection, and in the case of salt treatment, *TAS14* expression returned to basal levels within 24 hpi (**Figure 4B**).

The possibility of *P. capsici*-derived ABA was of interest because some plant pathogenic fungi produce ABA (Dorffling et al., 1984; Crocoll et al., 1991), and some stramenopiles such as the malarial pathogen, *Plasmodium falciparum*, are capable of ABA synthesis (Tonhosolo et al., 2009). However, we did not detect ABA in *P. capsici* culture filtrates or mycelium by immunoassay (Pye et al., 2013), and genes encoding the necessary biosynthetic enzymes are not evident in oomycete genomes (Tyler et al., 2006). Furthermore, we found no evidence that *P. capsici* infection further engages the pathway as part of its infection strategy, either in non-stressed or salt-stressed tomato plants. These results indicate that salt stress, but not *Phytophthora* infection, strongly engages the ABA pathway in tomato roots – *NCED1* and *TAS14* gene expression, and ABA synthesis and accumulation.

The SA-induced tomato PR protein, P4, is homologous to PR-1 in tobacco and *Arabidopsis. P4* gene expression is induced in tomato leaves by plant activators (SA-mimics), pathogens, including *Phytophthora infestans*, and the oomycete elicitor arachidonic acid (Joosten et al., 1990; Van Kan et al., 1992; Fidantsef et al., 1999). We found that infection of tomato roots by *P. capsici* strongly induces *P4*, but exposure of the roots to salt prior to inoculation essentially abolished *P4* expression relative to non-stressed, inoculated plants (**Figure 5**). Similarly, expression of the JA-induced *PI-2* was significantly reduced in infected plants that had been previously salt-stressed. Our findings that salt stress prevents pathogen-induced SA- and JA-regulated gene expression are consistent with results in other plant–microbe interactions that demonstrate ABA-mediated suppression of SA and JA defense responses (Anderson et al., 2004; Yasuda et al., 2008).

Tomato plants suppressed in SA accumulation by the *nahG* transgene are more susceptible to *P. capsici* than the wild-type control plants in both non-stressed and salt-stressed assay formats (**Figure 6**). This suggests a role for SA-mediated responses in partially limiting *P. capsici* colonization. However, the proportional increase in pathogen colonization observed in salt-stressed plants relative to non-stressed plants is the same in both WT and NahG backgrounds. Impairment of SA action by salt stress may contribute to increased pathogen colonization; however, we did not see a compounding effect of the SA-deficiency in NahG plants on stress-induced disease severity.

Salicylic acid’s role in tomato resistance to *P. capsici* is complex. In a study using chemical activators that mimic SA action to induce resistance, we found these activators when applied to roots induced systemic protection of tomato leaves against the bacterial speck pathogen (*Pst*), with and without predisposing salt stress (Pye et al., 2013). However, these same plant activator treatments afforded no protection against *P. capsici*, with or without the salt stress treatment. *Pst* and *P. capsici* are quite different in their infection strategies and requirements, as well as the organs they attack in the plant, so interpreting differences in disease outcomes following different treatments is a speculative exercise, at best. *P. capsici* may simply be a more aggressive pathogen relative to *Pst*, and our experimental format is highly conducive to root and crown rot disease. So *P. capsici* attack overwhelms any chemically induced resistance that is otherwise capable of withstanding *Pst* challenge. It is also possible that there is subfunctionalization within the SA response network in tomato. NahG expression may impair a set of SA-mediated defenses that are effective against *P. capsici*, but differ from a subset, induced by chemical activators, that are insufficient to resist this pathogen.

The JA-deficient tomato mutants *acx1* and *def1* in the ‘Castlemart’ background are compromised in defense against insects and pathogens (Ament et al., 2004; Bhattarai et al., 2008). Although severity of the predisposition phenotype can vary among tomato cultivars, we were astonished that ‘Castlemart’ and its JA mutants were not predisposed by salt, strongly trending instead toward enhanced resistance (**Supplementary Figure S3**). This suggests a stress response in ‘Castlemart’ that is different from other tomato genotypes we have examined in predisposition studies. The reason for this is unclear, and limited resources precluded our further examining predisposition in this cultivar. Unlike the other genotypes used in our study, ‘Castlemart’ is a processing variety with a pedigree that may have incorporated different stress tolerances. It is a determinate variety that was bred for arid climates, and arid zone soils are more commonly associated with salinity (R. Chetelat, personal communication). ‘Castlemart’ has been reported to accumulate proteinase inhibitors in response to high salinity (Dombrowski, 2003).

Jasmonic acid and its methyl ester when applied to leaves can induce resistance in tomato to *P. infestans* (Cohen et al., 1993). *Arabidopsis* mutants in JA perception (Staswick et al., 1998) and synthesis (Savchenko et al., 2010) are more susceptible to oomycete pathogens. Studies with other oomycete diseases also illustrate JA’s importance in resistance (Guerreiro et al., 2016). We found that exogenous JA enabled tomato roots to respond in a manner that partially offset the salt stress impairment of PR-protein gene expression (**Figures 7A,D**). The induction of *P4* only during infection of JA-treated plants is reminiscent of the reported sensitization by methyl jasmonate of the plant’s response to eicosapolyenoic acid elicitors released during infection by *Phytophthora* species (Il’inskaya et al., 2000) and potentiation of JA signaling by the plant activator β-aminobutryic acid (Hamiduzzaman et al., 2005).

Our results with the tomato genotypes and treatments used in this and previous studies (Dileo et al., 2010; Pye et al., 2013) affirms ABA’s dominant effect relative to the salt-induced impacts on SA and JA action during predisposition to Phytophthora root and crown rot. ABA appears to be necessary to predispose tomato seedlings to this disease following acute salt stress. However, results presented here and previously (Pye et al., 2013) indicate that priming through chemical activation (Ton et al., 2009a) of the SA and JA response networks may partially offset the stress-induced impairment of defense-related gene expression and the increased susceptibility in tomato to certain pathogens. We recognize that the response pathways modulated by ABA, JA and SA during episodic root stress may interact in subtle ways beyond the resolution afforded by the pathosystem and treatments we selected (Moeder et al., 2010). Comparative transcriptomics, proteomics and metabolomics of plants under predisposing stress should help identify key regulatory features (Bostock et al., 2014). Studies with additional mutants as well as salt- and drought-tolerant genotypes also may reveal additional variation that could be useful to refine our understanding of the abiotic-biotic stress ‘interactome’ (Pandey et al., 2015). This information could suggest novel targets to mitigate the impact of root stresses that increase severity of soilborne diseases.

## Author Contributions

Research conceived and planned by MP, JM, and RB, and executed by MP. Data were analyzed by MP and RB. Experiments presented in **Figure 7** were planned by RSR, SD, and RB, performed by RSR, and analyzed by RSR, SD, and RB. The manuscript was written by MP and RB. All authors approved the manuscript.

## Conflict of Interest Statement

The authors declare that the research was conducted in the absence of any commercial or financial relationships that could be construed as a potential conflict of interest.

## Supplementary Material

FIGURE S1 The salinity-induced predisposition phenotype in 4-week-old hydroponically grown ‘New Yorker’ tomato seedlings 48 hpi with *Phytophthora capsici* (10^4^ zoospores ml^-1^).

FIGURE S2 Confocal visualization of hyphae and haustoria (small arrow) of *P. capsici*-GFP in ‘New Yorker’ tomato root cells and nucleus (large arrowhead) stained with propidium iodide (PI), 48 hpi. Bars indicate 10 μm.

FIGURE S3 **(A)** *P. capsici* colonization 48 hpi on ‘Castlemart’ and JA- deficient *def1* (in ‘Castlemart’ background) tomato seedlings non-stressed (control) or salt stressed with 0.2 M NaCl/0.02 M CaCl_2_ for 18 h prior to inoculation. Colonization estimated by qPCR of pathogen DNA. Values are the means ± SE from three experiments, with five samples, each from a separate seedling, for each treatment within each experiment (*n* = 15). Differences are not significant (Wilcoxon rank sums test, χ^2^ = 3.08, *P* = 0.379). **(B)** *P. capsici* colonization 48 hpi on ‘Castlemart’ and JA-deficient *acx1* (in ‘Castlemart’ background) tomato seedlings non-stressed (control) or salt stressed with 0.2 M NaCl/0.02 M CaCl_2_ for 18 h prior to inoculation. Colonization estimated by qPCR of pathogen DNA. Values are the means ± SE from three experiments (*n* = 15) as in **(A)**. Asterisk indicates a significant difference between the *acx1* control and salt treatments (*T*-test, *P* = 0.032).
