# Supplementary figures and images for: Abscisic Acid as a Dominant Signal in Tomato During Salt Stress Predisposition to Phytophthora Root and Crown Rot
Source: Front Plant Sci. 2018 Apr 23;9:525. doi: 10.3389/fpls.2018.00525 (PMC5924805; doi:10.3389/fpls.2018.00525)

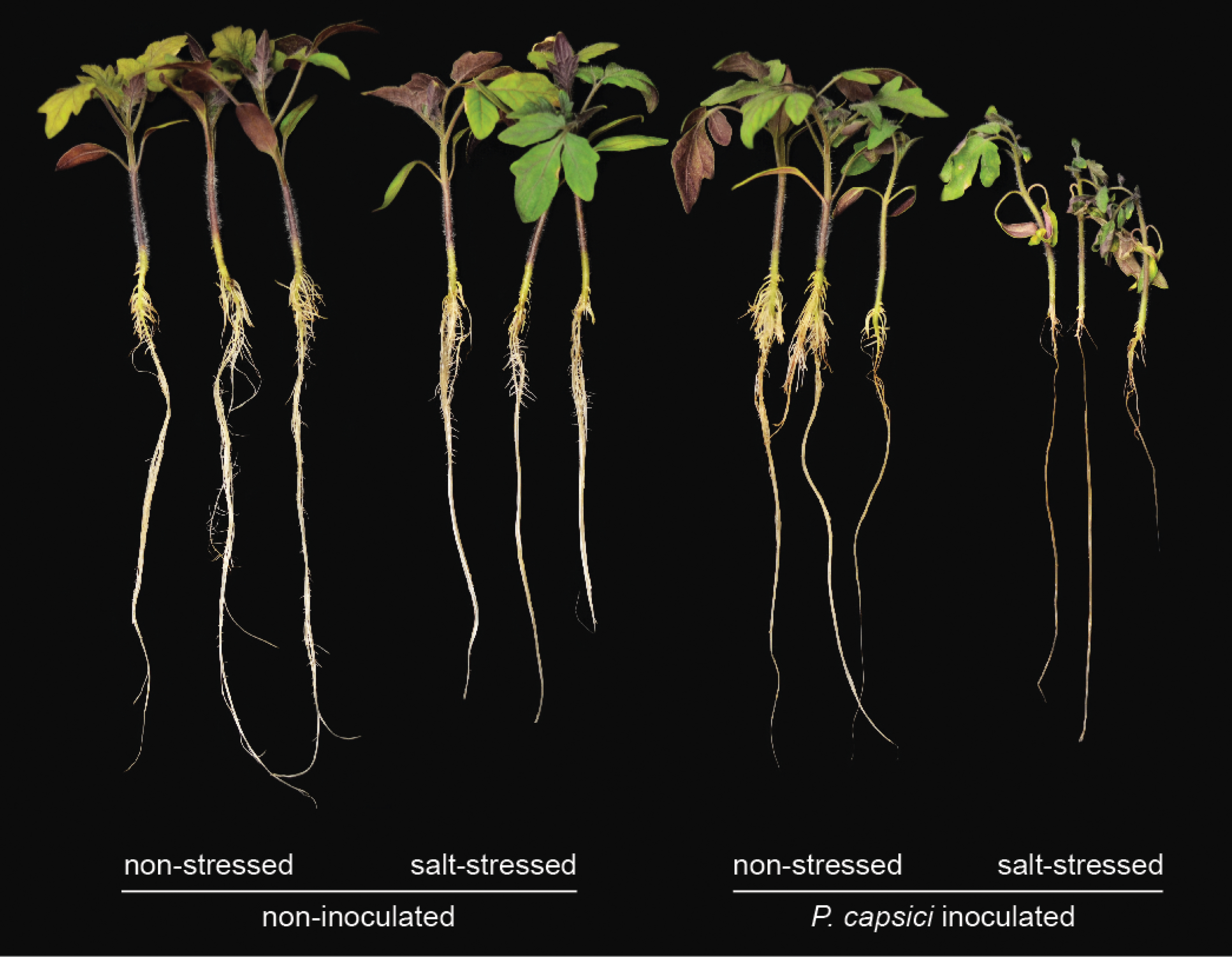

Supplement: FIGURE S1 — The salinity-induced predisposition phenotype in 4-week-old hydroponically grown ‘New Yorker’ tomato seedlings 48 hpi with Phytophthora capsici (104 zoospores ml-1). [file Image_1.JPEG]

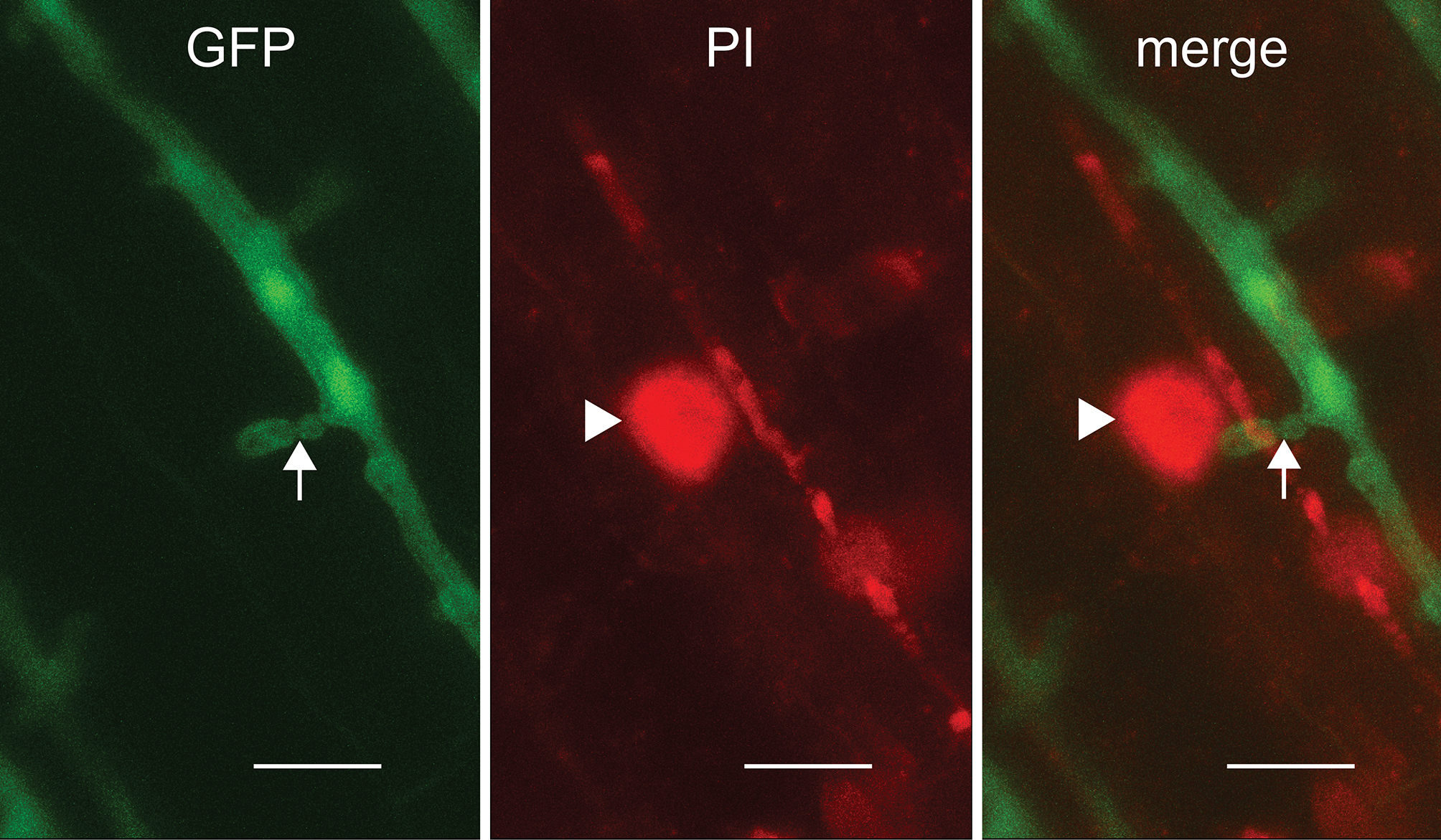

Supplement: FIGURE S2 — Confocal visualization of hyphae and haustoria (small arrow) of P. capsici-GFP in ‘New Yorker’ tomato root cells and nucleus (large arrowhead) stained with propidium iodide (PI), 48 hpi. Bars indicate 10 μm. [file Image_2.JPEG]

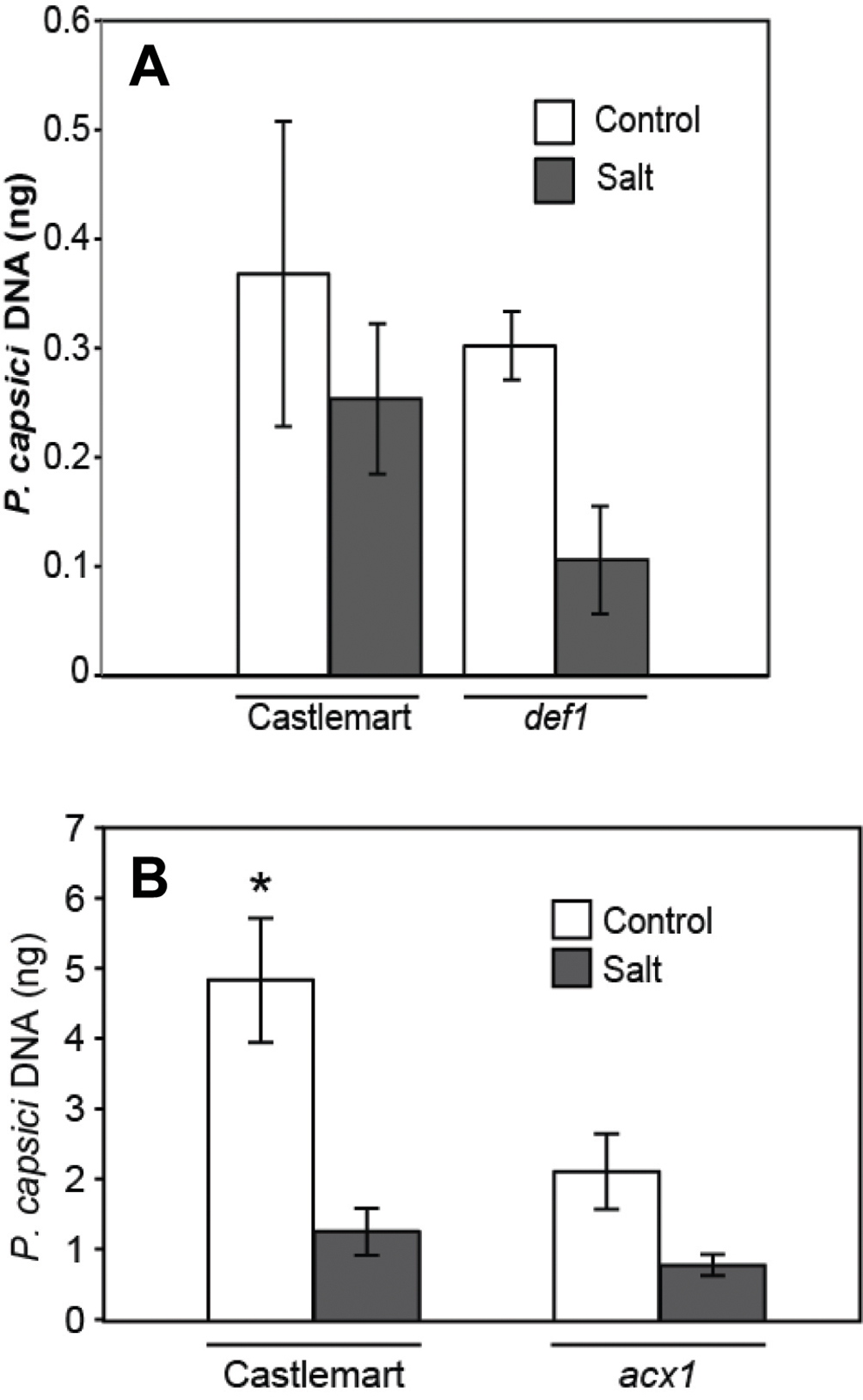

Supplement: FIGURE S3 — (A) P. capsici colonization 48 hpi on ‘Castlemart’ and JA- deficient def1 (in ‘Castlemart’ background) tomato seedlings non-stressed (control) or salt stressed with 0.2 M NaCl/0.02 M CaCl2 for 18 h prior to inoculation. Colonization estimated by qPCR of pathogen DNA. Values are the means ± SE from three experiments, with five samples, each from a separate seedling, for each treatment within each experiment (n = 15). Differences are not significant (Wilcoxon rank sums test, χ2 = 3.08, P = 0.379). (B) P. capsici colonization 48 hpi on ‘Castlemart’ and JA-deficient acx1 (in ‘Castlemart’ background) tomato seedlings non-stressed (control) or salt stressed with 0.2 M NaCl/0.02 M CaCl2 for 18 h prior to inoculation. Colonization estimated by qPCR of pathogen DNA. Values are the means ± SE from three experiments (n = 15) as in (A). Asterisk indicates a significant difference between the acx1 control and salt treatments (T-test, P = 0.032). [file Image_3.JPEG]
